# Evaluation of the reproductive system development and egg-laying performance of hens infected with TW I-type infectious bronchitis virus

**DOI:** 10.1186/s13567-020-00819-4

**Published:** 2020-07-31

**Authors:** Xiaorong Zhang, Kai Liao, Shuqin Chen, Kun Yan, Xubin Du, Chengcheng Zhang, Mengjiao Guo, Yantao Wu

**Affiliations:** Jiangsu Co-Innovation Center for the Prevention and Control of Animal Infectious Disease and Zoonoses, College of Veterinary Medicine, Yangzhou University, Yangzhou, 225009 Jiangsu People’s Republic of China

**Keywords:** infectious bronchitis virus, TW I-type, reproductive system development, egg-laying performance

## Abstract

The prevalence of TW I-type infectious bronchitis virus (IBV) has been increasing rapidly, and it has become the second most common genotype of IBV in China threatening the poultry industry. In this study, 1-day-old specific-pathogen-free (SPF) chickens infected with TW I-type IBV were continuously observed for 200 days. TW I-type IBV affected the respiratory, urinary, and female reproductive systems, resulting in a mortality rate of 10% as well as a decrease in egg quantity and an increase in inferior eggs. During the monitoring period, serious lesions occurred in the female reproductive system, such as yolk peritonitis, a shortened oviduct, and cysts of different sizes with effusion in the degenerated right oviduct. The infective viruses persisted in vivo for a long time, and due to the stress of laying, virus shedding was detected again after the onset of egg production. Our findings suggest that TW I-type IBV is deadly to chickens and could cause permanent damage to the oviduct, resulting in the poor laying performance of female survivors and decreasing the breeding value and welfare of the infected flock.

## Introduction

Avian infectious bronchitis is a highly contagious acute viral disease in chickens caused by infectious bronchitis virus (IBV) [1]. As a member of the genus *Gammacoronavirus*, IBV has a remarkably high mutation and recombination rate, leading to numerous types and variants that differ from each other in pathogenicity [2, 3]. Although the site of entry of IBV is the upper respiratory tract, where the initial infection occurs, the virus can spread systemically, replicating in the epithelial cells of many organs and causing injuries of the kidneys and female reproductive tract [4]. The kidney, trachea, caecal tonsil, and cloaca have been demonstrated to be tissues in which the long-term persistence of IBV is observed [5]. It has been reported that the virus can even be re-excreted from the faeces of H52-infected and H120-infected chickens at 227 days post-infection [6]. The mechanisms involved in the long-term persistence of virulent IBV in convalescent chickens are assumed to be related to viral pathogenicity, and further study is needed [7].

TW I-type IBV was first discovered in 1992 in Taiwan and subsequently identified as a new genotype. Since the first report in Chinese Mainland in 2009, the prevalence of this IBV type has increased rapidly nationwide [8]. It has been reported that QX-type strains accounted for 46.1% (95/206) of all isolates from 2013 to 2015 in southern China, with TW I-type strains being the second most prevalent (26.7%, 55/206) [9]. Due to differences in antigenicity, the protection provided by commercial vaccines in chickens infected with TW I-type IBV is not complete, and the incidence of immune failure caused by this strain type has increased in recent years [10]. The majority of TW I-type IBV strains exhibit widespread tissue tropism, and they can affect the respiratory, urinary and reproductive systems, and lead to the death of young chickens [11]. There is evidence suggesting that TW I-type viruses have undergone extensive evolution, with diverse strains circulating in chicken flocks, and the need to comprehensively evaluate the pathogenicity of this type of strain has become even more urgent [12, 13].

In this study, the pathogenicity of TW I-type IBV was evaluated by examining clinical symptoms, mortality rates, virus shedding, lesions, and laying performance in terms of egg quantity and quality in infected chickens. The aim of the study was to comprehensively reveal the pathogenicity of TW I-type IBV, particularly regarding the long-term impact on egg production.

## Materials and methods

### Viruses

Strain Ck/CH/AH/2011/3 (abbreviation: AH1103) of TW I-type IBV used in this study was isolated in 2011 from the trachea and kidney of chickens in a broiler flock exhibiting respiratory signs and death [14, 15]. AH1103 was serially diluted, and five replicate samples of 10^−3^, 10^−4^, 10^−5^, 10^−6^, and 10^−7^ dilutions were inoculated into 10-day-old embryonated SPF chicken eggs, the 50% egg infections dose (EID_50_) of this strain was calculated by the Reed–Muench method [16].

### Animals and ethics statement

SPF chicken eggs were purchased from the Beijing Boehringer Ingelheim Merial Vital Laboratory Animal Technology Co., Ltd, China. One-day-old SPF white leghorn chickens were purchased from the Jinan Sipai Furui Livestock Technology Co., Ltd. The operation and treatment of the animals were approved by the Institutional Animal Care and Use Committee of Yangzhou University (YZUDWLL-201902-001).

### Experimental design, observations and sampling

One-day-old SPF chickens (n = 140) were randomly divided into two groups, each group contained 70 chickens. At 30 days of age, all cockerels were picked out and discarded. The chickens were housed in separate negative-pressure isolators in biosafety level 2 facilities and supplied with feed and water ad libitum. At 1 day of age, the challenge group was infected via oculo-nasal route, with 100 μL of PBS diluent containing 10^5.5^ EID_50_ of the AH1103; and the control group was administered PBS instead. After challenge, the chickens were observed daily. The full trial period lasted 200 days.

The pathogenicity of TW I-type IBV in the early stage post-infection was evaluated via the clinical symptoms, pathological lesions, and virus shedding from trachea and cloaca. Dyspnea, gasping, tracheal rales, diarrhoea, depression, anorexia, and death in the infected chickens were recorded as clinical symptoms. At 5 days post-infection (dpi), five chickens in each group were euthanized by cervical dislocation for microscopic lesion examination. At 7 dpi and 14 dpi, the oral and cloaca swabs were collected from 10 chickens in each group for the detection of virus shedding by reverse transcription-quantitative polymerase chain reaction (RT-qPCR).

At 140 days of age, the effects of TW I-type IBV infection on the female reproductive system were evaluated. Nine hens of the challenge group and three hens of the control group were euthanized by cervical dislocation and necropsied. The developmental status of oviducts and ovaries were checked and documented. Then, fourteen chickens in each group were randomly chosen for comparison of egg production performance and egg quality after the onset of laying.

In the laying period, the eggs were collected daily, and quality traits were evaluated for each egg in the next morning [17]. Eggs were weighed individually, and their egg weight recorded to the nearest 0.1 g. Egg length and width was measured to the nearest 0.1 cm using calipers. The width was divided by the length and multiplied by 100 to obtain the shape index. Eggs were broken onto a flat surface and the height of the albumen was measured in millimeters using a tripod micrometer halfway between the edge of the yolk and thick albumen. The thickness of the eggshell was measured in 3 places around the midline to the nearest 0.01 mm, and averaged. At 156 days of age, the oral and cloaca swabs were collected from 6 hens in each group for the detection of virus re-excretion by virus isolation and RT-qPCR. At 200 days of age, all of the hens were euthanized by cervical dislocation and necropsied. The samples of trachea, lungs, kidneys, oviduct, heart, liver, spleen, cecal tonsils, duodenum, and glandular stomach of 6 chickens were collected, and tissues from every two chickens were pooled for viral RNA detection by RT-qPCR.

### Virus isolation and RT-qPCR

The oral and cloaca swabs were washed in PBS, and suspensions of tissues were also prepared in PBS (20% w/v). Samples of 156-day-old hens were blind passaged three times in the allantoic cavity of 10-day-old embryonated SPF chicken eggs. Total RNA of the samples were extracted using the Ultrapure RNA Kit (CoWinbio, Beijing, China), and reverse transcription was performed with the EasyScript^®^ Reverse Transcriptase [M-MLV, RNaseH-] Kit (TransGen Biotech, Beijing, China). The primers and probe used for RT-qPCR were described in a previous study [18]. The reaction mixture and the thermal profile employed for RT-qPCR were as specified in the AceQ qPCR Probe Master Mix Kit (Vazyme Biotech, Nanjing, China).

### Histopathology

For haematoxylin–eosin staining, the collected samples of the trachea, lungs, kidneys, and oviduct were fixed in 10% neutral formalin for 48 h at room temperature. The fixed samples were processed, embedded in paraffin wax, and cut into 5 μm sections and examined by light microscopy for the presence of lesions.

For the examination of ultrastructural alterations, the cylindrical trachea were cut crosswise with two sharp blades into small strips of 1 mm wide and 3–4 mm long, and then the strips were cut into 1 mm^2^ small squares. The squares were then fixed in 2.5% glutaraldehyde in 0.1 M PBS for 12 h at 4 °C. The samples were then washed with PBS and dehydrated via an alcohol gradient, followed by drying (CPD-30D) and conductive treatment (SCD 500). Finally, the ultrastructure of the tracheal cilia was observed by scanning electron microscopy (GeminiSEM 300) with a magnification of 5000×.

### Statistical analysis

All statistical analyses were performed with SPSS 26.0 software. Non-parametric Mann–Whitney U test was used to analyze the significant difference, and *p *< 0.05 was considered as significant.

## Results

### Clinical manifestations

The clinical manifestations in the early infection stage caused by TW I-type IBV were identified as respiratory symptoms. Nearly half of the chickens in the challenge group exhibited gasping, tracheal rales or dyspnea. Seven chickens died from 9 to 30 dpi, accounting for 10% of the infected flock. Gross lesions such as light hyperaemia and serious catarrhal exudation in the tracheal mucosa and urate deposition in the tubules of the pale kidney, appeared in the infected flock from 5 to 30 dpi.

**Figure 1:**
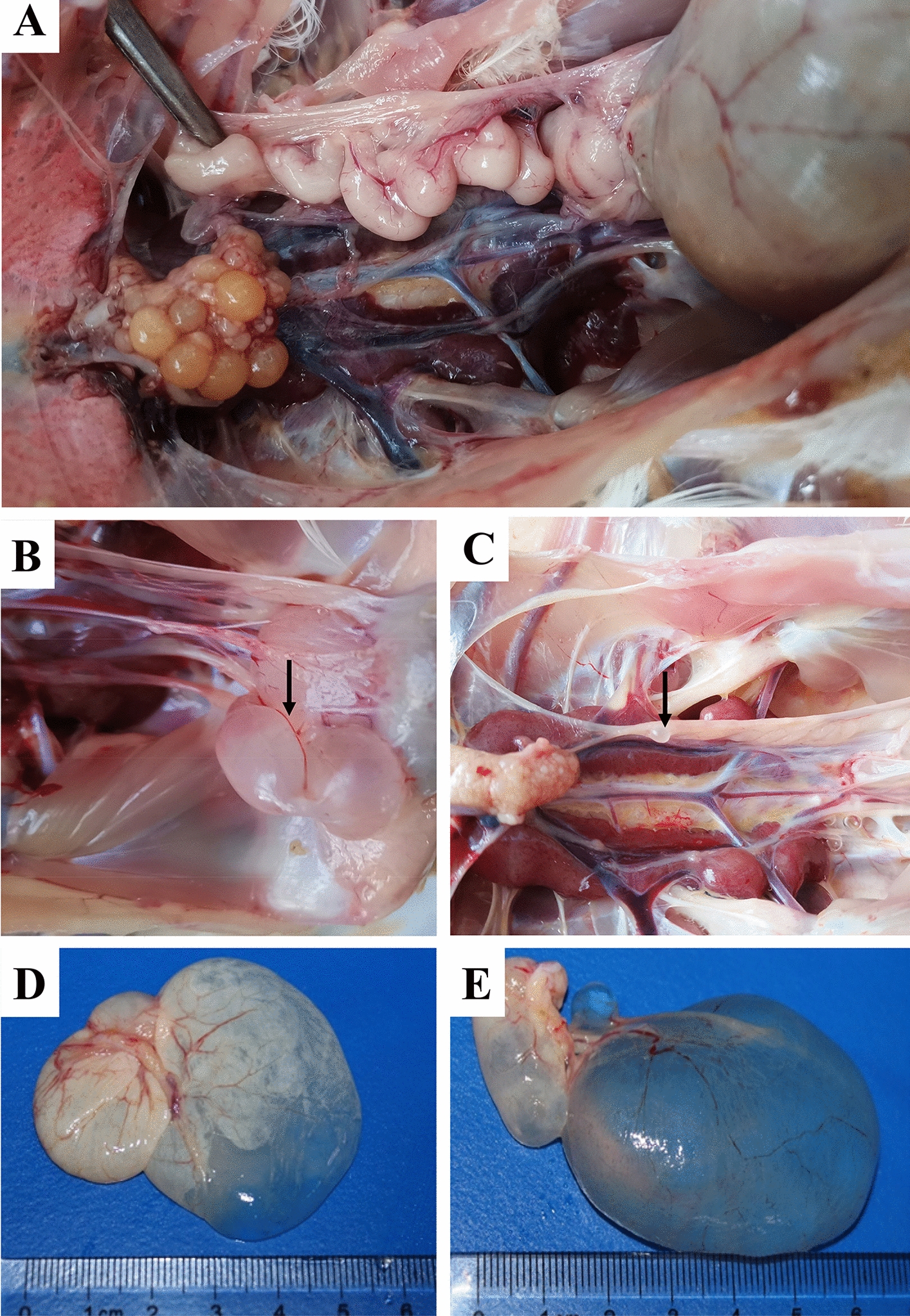
**Cystic dilatation of the oviduct. A** Well-developed oviduct of a 140-day-old chicken in the control group. **B**, **C** Oviduct cysts and developmental delay of the reproductive system in chickens from the challenge group at 140 days of age. **D**, **E** Oviduct cysts in chickens from the challenge group at 200 days of age.

The development of oviducts and ovaries was assessed at 140 days of age. The reproductive system of chickens in the control group was well developed. However, there were 4/9 chickens in the challenge group that showed delayed development of oviducts and ovaries (Table 1). The right oviducts of all chickens in both control and challenge groups were completely degenerated. Right cystic dilatations attached to the cloaca with a watery content were present in 5/9 of the chickens from the challenge group, and another 1/9 chicken had a cyst on the left immature oviduct wall (Table 1, Figure 1).

**Table 1:** **Incidence of oviduct cysts and the developmental delay of the reproductive system caused by TW I-type IBV**

Group	Oviduct cyst		Stunted oviduct		Stunted ovary	
	140 dpi	200 dpi	140 dpi	200 dpi	140 dpi	200 dpi
Challenge	6/9	10/14	4/9	0/14	4/9	0/14
Control	0/3	0/14	0/3	0/14	0/3	0/14

**Figure 2:**
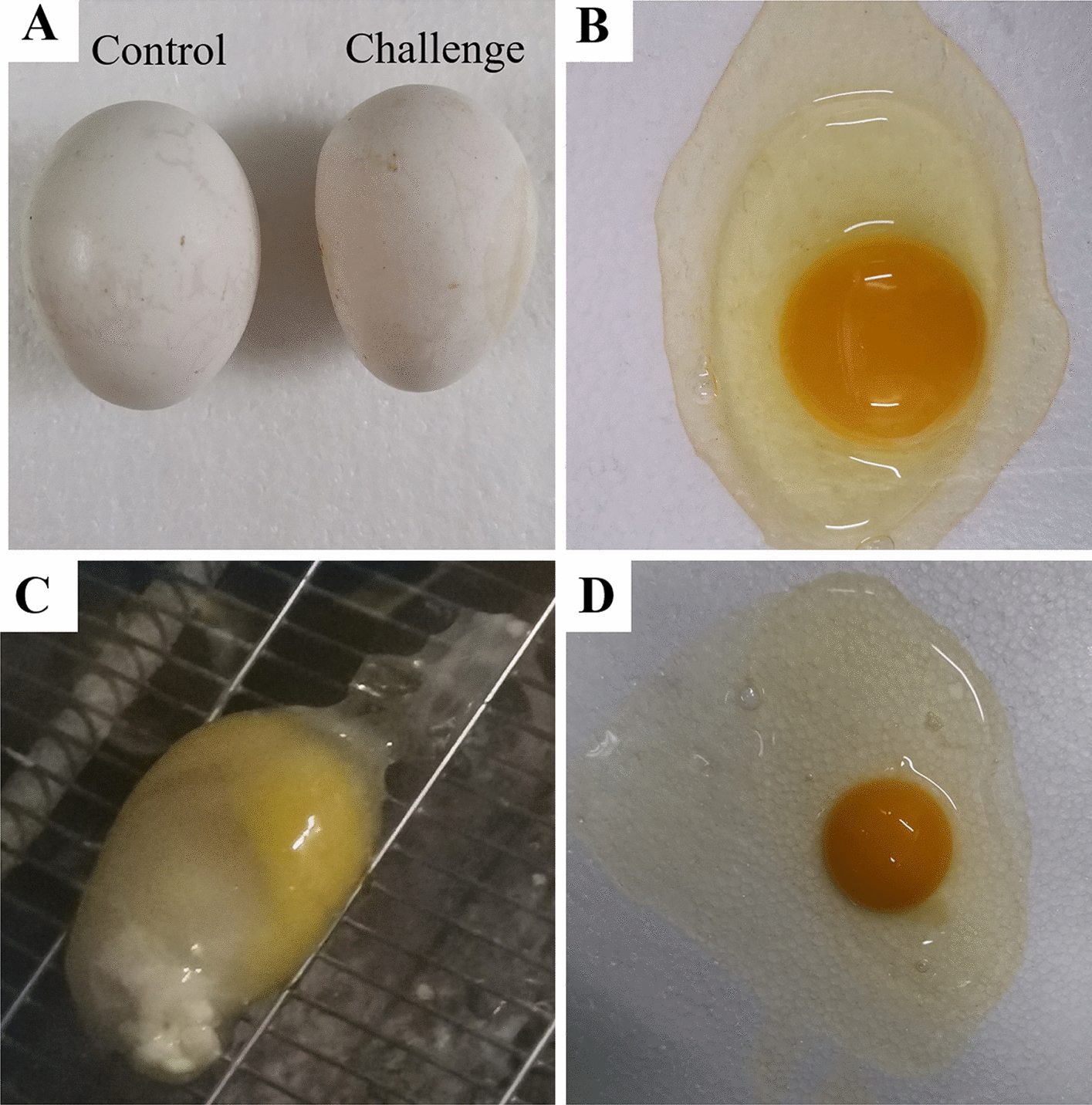
**Impacts of TW I-type IBV on egg quality in chickens infected at 1** **day of age. A** A normal egg from the control group (left) and misshapen egg from the challenge group (right). **B** Egg with normal albumen from the control group. Shell-less egg (**C**) and egg with watery albumen (**D**) from the challenge group.

The pathogenicity of TW I-type IBV in the laying period was characterized by a decrease in the quantity of eggs and an increase in the quantity of inferior eggs (Table 2, Figure 2). During the 21.5- to 27.5-week-old in which laying was monitored, egg production in the challenge group was lower than that in the control group; the decrease of egg production was 19.3% in the challenge group; and the peak egg production per week (66.3%) was lower than that in the control group (85.7%). The mean albumen height in the challenge group was 0.82 mm thinner than that in the control group, representing a decrease of 10.39% (*p *< 0.05). These results indicate that TW I-type IBV has a significant effect on subsequent laying performance when the infection occurs at a very young age in chickens.

**Table 2:** **The impacts on egg quality caused by TW I-type IBV infection at 1** **day of age**

Group	Egg production	Peak laying rate per week (%)	Egg weight (g)	Shape index	Albumen height (mm)	Shell thickness (mm)	Misshapen egg	Watery albumen	Shell-less egg
Challenge	257^a^	66.30	52.77 ± 4.48	76.55 ± 6.21	7.07 ± 1.51^a^	0.4 ± 0.04	6/257	15/257	1/257
Control	392^b^	85.70	52.94 ± 5.03	75.38 ± 4.12	7.89 ± 1.85^b^	0.38 ± 0.04	0/392	0/392	0/392

^a,b^ Values with different superscripts differ significantly (*p *< 0.05).

**Figure 3:**
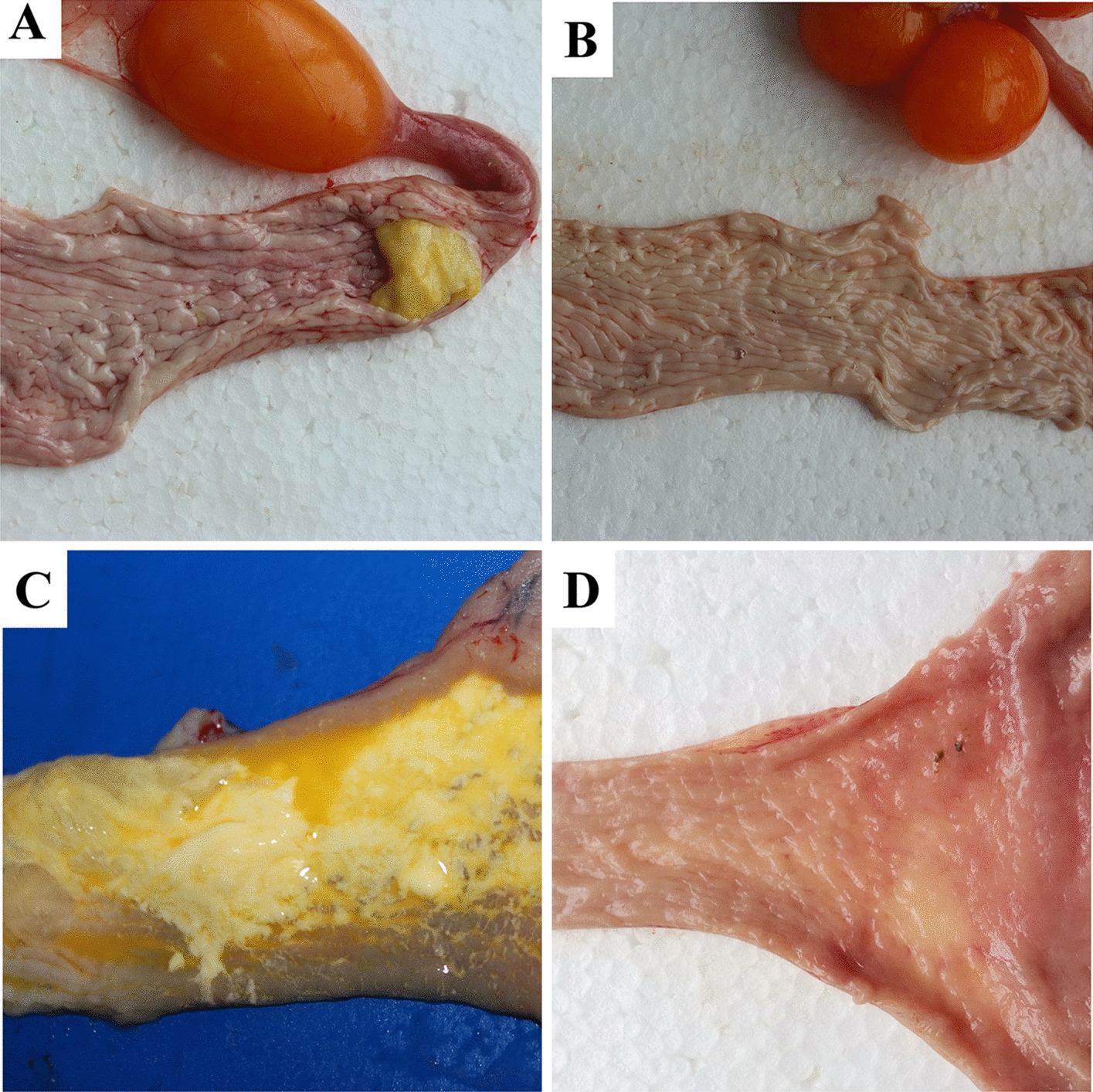
**Fibrin clot in oviduct at 200** **days. A**, **C** Fibrin clots (yolk-like) in the magnum and uterus of the infected hens, respectively. **B**, **D** Oviducts from hens of the control group.

At the age of 200 days, various lesions were observed in the reproductive systems of the hens (Table 3, Figure 5). In the challenge group, lesions of yolk peritonitis were found in 7 infected females, which were assumed to have been caused by the mature follicles or eggs falling into the abdomen and failing to be captured (Table 3). Almost all of the females exhibited a large or small oviduct cyst, three of which presented a diameter greater than 3 cm (Figure 1). A fibrin clot (yolk-like) in the lumen of the oviduct was observed in 3 females (Figure 3). None of these lesions were found in the control group.

**Table 3:** **The lesions of oviducts caused by TW I-type IBV at 200** **dpi**

Group	Fibrin clot in oviduct	White spots on oviduct	Yolk peritonitis	Necrotic follicle in the ovary	Thin uterus wall^a^	Number of follicles^b^	Length of oviduct^c^
Challenge	3/14	6/14	7/14	1/14	1/14	4.4 ± 1.5	66.4 ± 6.7
Control	0/14	0/14	0/14	0/14	0/14	5.5 ± 0.5	73.0 ± 3.5

^a^ “Thin” uterus wall means that the uterine wall was thin and that there were few folds in the uterus.

^b^ The follicles which a diameter of more than 1.5 cm in the ovary were recorded.

^c^ It is the total length (cm) of the infundibulum, magnum, and isthmus.

### Virus shedding

**Figure 4:**
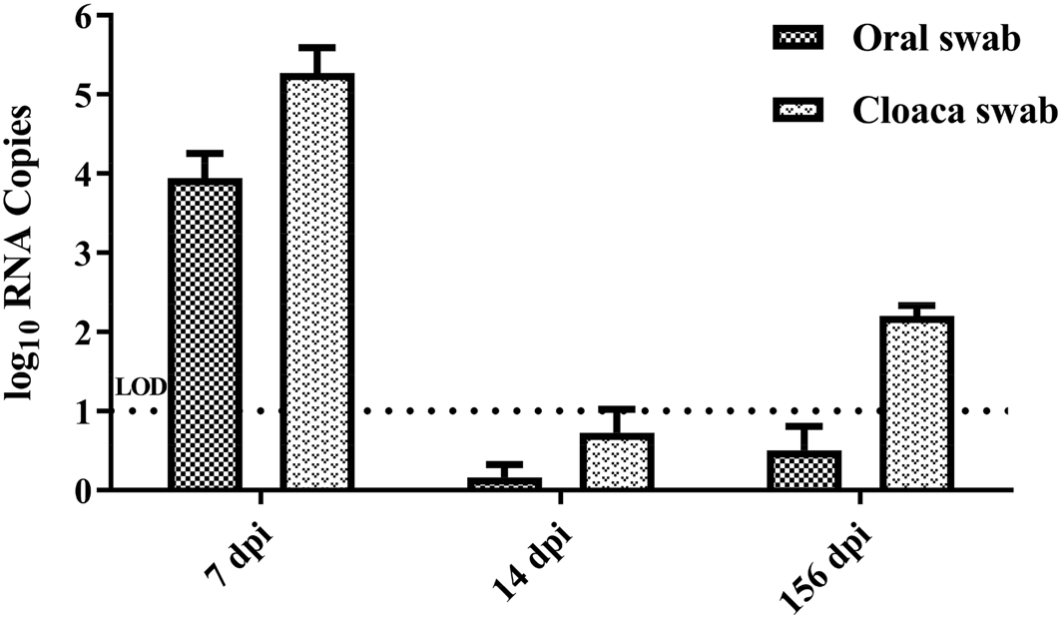
**Detection of viral RNA by RT-qPCR assay.** The results of virus shedding from the trachea and cloaca at 7, 14, and 156 dpi. The dotted line represented the limit of detection (LOD).

The RT-qPCR results for the oral and cloaca swabs collected at 7 dpi and 14 dpi indicated virus shedding via the respiratory tract and cloaca at 7 dpi (Figure 3). At 156 days of age, the viruses were re-excreted from the cloaca (2/6 in the challenge group), about 10^2.2^ copies/μL, and two positive samples were blind passaged three times in SPF chicken eggs, 10^6.7^ and 10^8.3^ copies/μL were detected. At 200 dpi, one of the pooled caecal tonsil samples was detected containing 10^4.1^ copies/μL (Figure 4).

### Histopathological lesions

**Figure 5:**
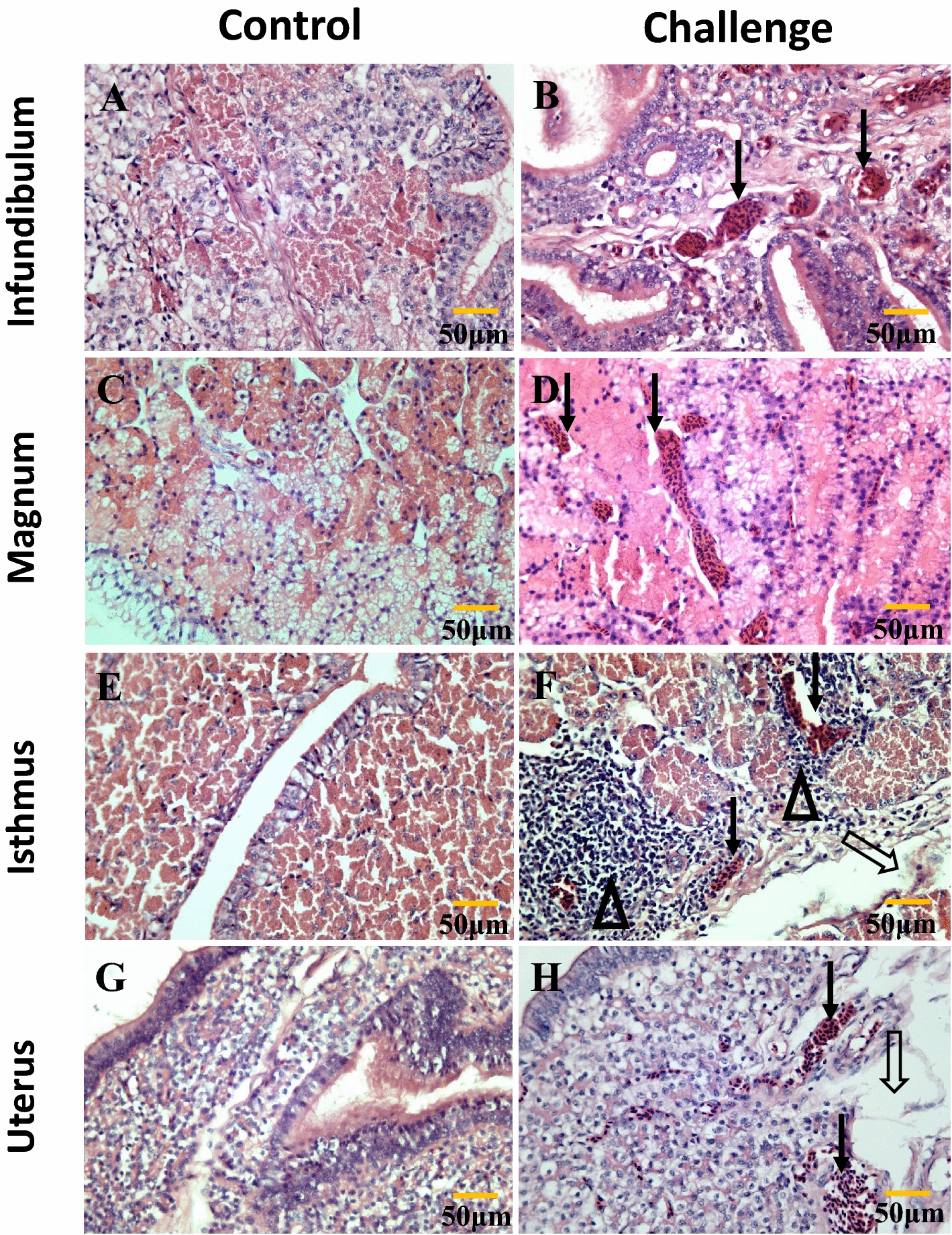
**Histopathology of the oviduct at 200** **days. A**, **C**, **E** and **G** are controls. **B** (infundibulum): solid arrows indicate congestion in the submucosa. **D** (magnum): the solid arrow indicates congestion in the lamina propria and broadening of the interstitial region. **F** (isthmus): the hollow arrow indicates desquamation of epithelial cells; solid arrows indicate congestion in the submucosa; hollow triangles indicate lymphocytic infiltration. **H** (uterus): the hollow arrow indicates edema in the lamina propria; solid arrows indicate congestion in the lamina propria.

The ultrastructural examination showed the presence of catarrhal exudates and the adhesion, lodging, and shedding of cilia on the tracheal surface of the infected flock at 5 dpi (see Additional file 1). The tracheae were collected at 5 dpi; the lesions of the blurred boundary between the cilia, congestion, inflammatory cell infiltration, and necrosis of ciliated epithelial cells were widespread. In the laying period, the lesions of the congestion, inflammatory cell infiltration, broadening of the interstitial region, and desquamation of epithelial cells in the oviducts were common in the infected flock (Figure 5). No significant lesions were observed in the control group.

## Discussion

The prevalence of TW I-type IBV has increased greatly in China in recent years, which has caused tremendous economic losses to the poultry industry [9]. Some reports have illustrated that the majority of TW I-type IBV show tissue tropism to the kidneys and trachea and result in a moderate mortality rate [19, 20]. However, there are no long-term reports on the effects of TW I-type IBV on the quantity and quality of eggs when infection occurs at a very young age in chickens, although the poor laying performance and occurrence of “false layers” caused by QX-type IBV have attracted widespread attention [21, 22]. In this study, a 200-day pathogenicity study of the TW I-type IBV strain in chickens was performed. The results suggest that this strain can result in death and has consecutive adverse effects on the female reproductive system, which decrease the breeding value and welfare of the infected flock.

In the early infection stage, the pathogenicity of TW I-type IBV in chickens is similar to that of other prevalent types of strains regarding, for example, the symptoms of tracheal rales, and dyspnea or lesions of urate deposition in the kidneys [22, 23]. In terms of the ultrastructural and microstructural examination of the trachea, the lesions of adhesion and lodging and the shedding of tracheal cilia caused by TW I-type IBV are assumed to result directly from inflammatory cell infiltration and the presence of serious catarrhal exudates [24]. Respiratory lesions, which are the most common pathogenic characteristic of IBV, subsequently recover and do not lead to the death of infected chickens [25, 26]. Similar to QX-type strains, the renal lesions of urate deposition may be responsible for the death of chickens, although the mortality rate caused by TW I-type IBV was relatively low [27, 28].

During the laying period, the impacts caused by TW I-type IBV in females can be summarized into four categories: a high incidence of oviduct cysts, consecutive lesions in the female reproductive system, decreases in egg production, and poor quality of eggs. The mechanisms involved may contribute to the lesions in the reproductive system that emerged throughout the monitoring period and were induced by the virus reserves in the caecal tonsil. Some previous studies have illustrated that IBV exhibits reproductive tissue tropism and that the virus induces an immune response involving an influx of cytotoxic cells and upregulation of inflammatory cytokines in the oviduct and ovary [29, 30]. According to a previous report, the two main candidate sites for IBV persistence are cecal tonsils and the kidneys [31]. However, in this study, we found that the cecal tonsil was the only site of virus replication at 200 days of age. In addition, the exacerbated inflammation and physiological disorders in the oviduct caused by reinfection would be further worsened due to the effect of oestrogen [32]. As a result, during the laying period, the congestion and inflammatory cell infiltration in the oviducts were more severe than at any earlier time. These adverse impacts were subsequently reflected in a series of lesions in the female reproductive system, such as shortening of the length of the oviduct and a decrease in the number of hierarchical follicles in the ovary, which were directly responsible for a portion of the decrease in egg production. However, the pathogenicity of IBV in the female reproductive system is irregular, and the factors involved include the strains of the virus, the age of the infected chickens, and the protection afforded by antibodies [21, 33]. Some QX-type strains can also induce oviduct cysts with fluid accumulation in chickens of different ages, but some 4/91-type strains fail to do so [34, 35]. Not all Mass-type strains can induce cystic oviducts or the occurrence of false layers, which differ in different strains [26, 35]. Some recombinant strains can also induce oviduct cysts even when chickens have been inoculated with commercial vaccines [33]. The long-term decrease in egg production and the shortened oviduct observed at 200 dpi suggest that the damage to the reproductive system caused by TW I-type IBV might be a permanent impairment rather than just a developmental delay [36].

In conclusion, TW I-type IBV is characterized by respiratory symptoms, urate deposition lesions in the kidneys, and the continuous disturbance of the female reproductive system, resulting in a high incidence of oviduct cysts and a decline in the quantity and quality of eggs.

## Supplementary information

**Additional file 1. Ultrastructure of the tracheal surface at 5** **dpi observed by scanning electron microscope.** A is the control. B (challenge): adhesion, lodging of cilia covered by mucus. C (challenge): shedding and remains of cilia.

## Data Availability

The datasets analyzed during the current study are available upon request from the corresponding authors.

## Abbreviations

IBV: infectious bronchitis virus
SPF: specific-pathogen-free
dpi: days post infection
RT-qPCR: reverse transcription-quantitative polymerase chain reaction
