# Supplementary figures and images for: Evaluation of the reproductive system development and egg-laying performance of hens infected with TW I-type infectious bronchitis virus
Source: Vet Res. 2020 Jul 31;51:95. doi: 10.1186/s13567-020-00819-4 (PMC7393890; doi:10.1186/s13567-020-00819-4)

## Slide 1
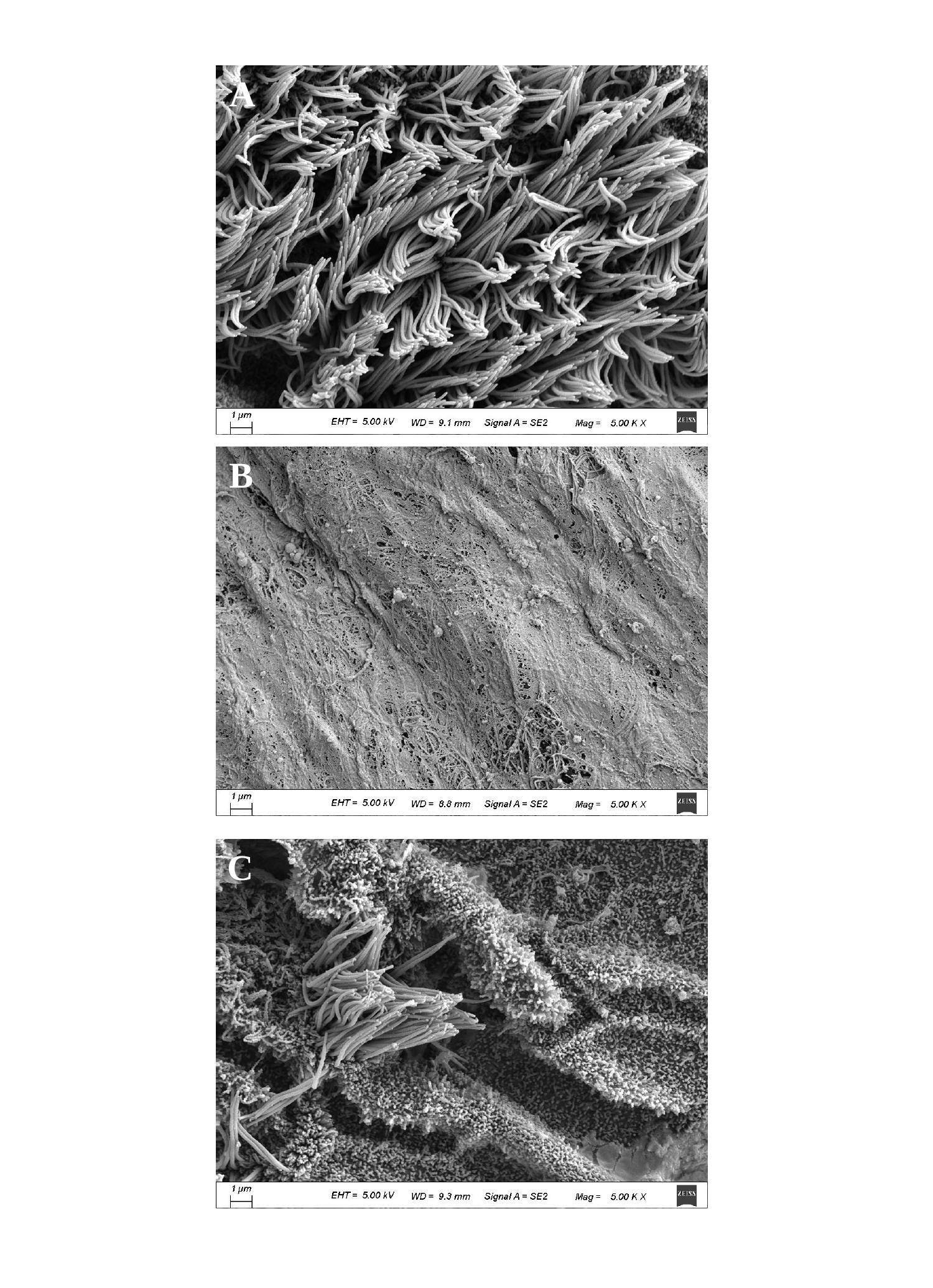

A
B
C

Supplement: Supplementary file 1 — Additional file 1. Ultrastructure of the tracheal surface at 5 dpi observed by scanning electron microscope. A is the control. B (challenge): adhesion, lodging of cilia covered by mucus. C (challenge): shedding and remains of cilia. [file 13567_2020_819_MOESM1_ESM.pptx]
